# Kidney Function Trajectories in Patients with and Without Chronic Kidney Disease Undergoing Transcatheter Aortic Valve Replacement: A Single-Retrospective Cohort Study

**DOI:** 10.3390/jcm15197656

**Published:** 2026-10-02

**Authors:** Hirofumi Sakuma, Satoshi Kawaguchi, Yuya Kitani, Tatsuya Aonuma, Naoko Kawabata, Erika Saito, Akiho Minoshima, Shingo Kunioka, Hiroyuki Kamiya, Naoki Nakagawa

**Affiliations:** 1 Division of Cardiology and Nephrology, Department of Internal Medicine, Asahikawa Medical University, Midorigaoka-Higashi 2-1-1-1, Asahikawa 078-8510, Japan; hsakuma210@asahikawa-med.ac.jp (H.S.); s-kawa@asahikawa-med.ac.jp (S.K.); ykitani@asahikawa-med.ac.jp (Y.K.); aonu@asahikawa-med.ac.jp (T.A.); naoko070@asahikawa-med.ac.jp (N.K.); erysaito@asahikawa-med.ac.jp (E.S.); minocy@asahikawa-med.ac.jp (A.M.); 2 Department of Integrative Nephrology and Therapeutics, Asahikawa Medical University, Midorigaoka-Higashi 2-1-1-1, Asahikawa 078-8510, Japan; 3 Department of Cardiac Surgery, Asahikawa Medical University, Midorigaoka-Higashi 2-1-1-1, Asahikawa 078-8510, Japan; s_kunioka@asahikawa-med.ac.jp (S.K.); kamiya@asahikawa-med.ac.jp (H.K.)

**Keywords:** transcatheter aortic valve replacement, chronic kidney disease, estimated glomerular filtration rate slope

## Abstract

**Background/Objectives**: Transcatheter aortic valve replacement (TAVR) may improve systemic hemodynamics and kidney perfusion; however, its effect on long-term kidney function remains unclear. We evaluated kidney function trajectories before and after TAVR in patients with and without chronic kidney disease (CKD). **Methods**: This single-center retrospective cohort study included patients with severe aortic stenosis (AS) who underwent TAVR between June 2016 and December 2024. Overall, 314 eligible patients were classified by baseline estimated glomerular filtration rate (eGFR) as without CKD (≥60 mL/min/1.73 m^2^) or with CKD (<60 mL/min/1.73 m^2^). Linear mixed-effects models estimated pre- and post-TAVR eGFR slopes, which were compared between groups after covariate adjustment. **Results**: Median follow-up was 2.0 years. Adjusted pre-TAVR eGFR slopes were −2.11 (95% confidence interval [CI], −3.34 to −0.89) mL/min/1.73 m^2^/year in patients without CKD and −2.83 (95% CI, −3.65 to −2.01) mL/min/1.73 m^2^/year in patients with CKD. The post-TAVR slopes were −4.02 (95% CI, −5.68 to −2.35) mL/min/1.73 m^2^/year in patients without CKD and −0.83 (95% CI, −2.04 to 0.39) mL/min/1.73 m^2^/year in patients with CKD, with a significant group difference of 3.19 (95% CI, 1.44 to 4.94) mL/min/1.73 m^2^/year (*p* < 0.001). In patients with CKD, the post-TAVR slope improved relative to the pre-TAVR slope by 2.01 (95% CI, 0.51 to 3.50) mL/min/1.73 m^2^/year (*p* < 0.01), whereas no significant change was observed in those without CKD. **Conclusions**: eGFR decline after TAVR was attenuated in patients with CKD compared with those without CKD, suggesting that kidney function may be preserved through hemodynamic improvement following TAVR.

## 1. Introduction

Aortic stenosis (AS) is the most common, progressive valvular disease in high-income countries, with a prevalence of 1–4% in adults aged >65 years [1,2,3]. In Japan, a recent epidemiological study estimated that the prevalence increased from 1.83% in individuals aged 60–64 years to 9.27% in those aged ≥85 years [4]. With the global population aging, the number of older adults with calcific AS is expected to more than double by 2050 in both Europe and the United States [5]. The prevalence of AS continues to increase owing to population aging worldwide, making it a significant issue and healthcare burden. Although AS often remains asymptomatic, several critical events, such as syncope, heart failure, and sudden death, occur with progression of valvular stenosis [6]. In patients with symptomatic severe AS, the associated morbidity escalates significantly, with 1- or 2-year mortality approaching 50% without valve intervention [6,7]. The standard interventional strategy for severe AS is aortic valve replacement (AVR), which comprises surgical AVR (SAVR) and transcatheter AVR (TAVR) [6]. The choice between SAVR and TAVR is individualized based on age, comorbidities such as coronary artery disease and chronic kidney disease (CKD), perioperative risk, and patient preferences [8,9]. TAVR is now an established option for older patients and those with a greater comorbidity burden who require aortic valve intervention.

AS is frequently observed among patients with CKD because of progressive, accelerated calcification [10]. Long-standing AS may contribute to CKD by impairing forward blood flow from the heart, causing chronic hypoperfusion and resulting in organ damage. Therefore, a valve replacement strategy for AS can reverse this pathophysiological mechanism of CKD [10,11]. Some studies showed that hemodynamic benefits, including improved cardiac output and reduced vascular tone and venous pressure after successful treatment of AS, have the potential to improve kidney function [11,12]. Conversely, other studies showed that 10–30% of patients after TAVR developed acute kidney injury (AKI) during the TAVR procedure, in the setting of contrast media administration, short periods of hypotension due to rapid pacing and balloon valvuloplasty, and large-bore catheter manipulation in the aorta of patients at risk for cholesterol embolization [13,14,15]. Although AKI after TAVR has been relatively well described, studies evaluating long-term changes in kidney function after TAVR remain limited. Therefore, the trajectory of kidney function following TAVR remains controversial.

Given the potential reversibility of CKD pathophysiology after correction of aortic valve disease, this study aimed to evaluate changes in kidney function after TAVR and determine whether the kidney response to TAVR differs according to CKD status. Given the growing evidence supporting estimated glomerular filtration rate (eGFR) decline as a surrogate endpoint for kidney disease progression and subsequent kidney failure [16,17], we analyzed pre- and post-TAVR eGFR slopes using linear mixed effects models and compared them between patients with and without CKD.

## 2. Materials and Methods

### 2.1. Study Design and Population

This retrospective cohort study was conducted at Asahikawa Medical University, a single tertiary medical center. Patients with severe AS who underwent TAVR between 1 June 2016 and 31 December 2024 were eligible. Severe AS was diagnosed by echocardiographic findings, and eligibility for TAVR was determined after multidisciplinary discussion by our heart team, consisting of cardiologists, cardiothoracic surgeons, echocardiographers, and anesthesiologists. The study period began in June 2016, when the first TAVR procedure was performed at our institution, and extended through December 2024 to allow sufficient post-TAVR assessment. The exclusion criteria were as follows: (1) end-stage kidney disease (eGFR < 15 mL/min/1.73 m^2^) or suspected overestimation (eGFR ≥ 100 mL/min/1.73 m^2^) on the day of admission, and (2) no eGFR measurement ≥ 30 days after TAVR, irrespective of the number of eGFR measurements available within the first 29 days after TAVR. These criteria were established to more accurately characterize the post-TAVR eGFR trajectory. This study was approved by the Asahikawa Medical University Research Ethics Committee (approval no. 25084). The requirement for informed consent was waived because of the retrospective nature of the study; an opt-out approach was used.

### 2.2. Transcatheter Aortic Valve Replacement Procedures

The procedural plan was discussed and finalized after preoperative systematic evaluation by the heart team. During the preoperative systematic evaluation, all patients underwent transthoracic or transesophageal echocardiography; coronary angiography; contrast-enhanced or non-contrast computed tomography; assessment of aortic annular dimensions; and vascular access evaluation of the cardiac apex, ascending aorta, common carotid arteries, subclavian arteries, and iliofemoral arteries. Severe AS was defined as an aortic valve area (AVA) < 1.0 cm^2^ or an indexed AVA < 0.6 cm^2^/m^2^, a mean transaortic pressure gradient ≥ 40 mmHg, and a maximum velocity ≥ 4.0 m/s on echocardiography. TAVR procedures were performed according to standardized techniques. Prosthesis size was determined from echocardiographic assessments and multislice computed tomography findings, based on current commercial recommendations. All patients underwent TAVR using the SAPIEN (SAPIEN 3 or SAPIEN3 Ultra RESILIA; Edwards Lifesciences, Irvine, CA, USA) or the Evolut (Evolut PRO+ or Evolut FX; Medtronic, Minneapolis, MN, USA). The optimal access route was selected by the heart team based on the patient’s anatomical and clinical characteristics from the transfemoral, trans-left subclavian, trans-right or trans-left common carotid, trans-ascending aorta, or trans-apex approaches. Because contrast-induced acute kidney injury (AKI) may occur frequently in older patients and in the presence of complications, intravenous saline infusion at 0.5–1.0 mL/kg/h was administered during the perioperative period. After the TAVR procedure, patients who were not already receiving antiplatelet therapy were prescribed at least one oral antiplatelet agent consisting of aspirin (100 mg, once daily) and/or clopidogrel (75 mg, once daily).

### 2.3. Data Collection

Clinical data obtained on the day of admission for the TAVR procedure, including age, sex, blood pressure, body weight, laboratory data, comorbidities, echocardiographic data, and medication use, were extracted from the institutional electronic medical record system. The eGFR value for each patient was calculated from serum creatinine using the Japanese eGFR equation as follows: eGFR (mL/min/1.73 m^2^) = 194 × serum creatinine (−1.094) × age (−0.287) × 0.739 (if female) [18]. CKD was defined as eGFR < 60 mL/min/1.73 m^2^ on the day of admission. Patients were categorized as having CKD or not having CKD based on eGFR data obtained on the same day. The index day was defined as the day of TAVR. The pre- and post-TAVR periods were defined as the duration before and after the index day, respectively.

### 2.4. Outcomes

The study endpoint was the difference in kidney function trajectories between the pre- and post-TAVR periods. Trajectories were evaluated in three complementary ways: (1) absolute eGFR values over time, (2) changes in eGFR from the index day at each measurement point (defined as ΔeGFR), and (3) eGFR slopes estimated using simple linear regression or linear mixed-effects models. Trajectories were compared between groups. Additionally, the kidney outcome was defined as two consecutive eGFR measurements of <15 mL/min/1.73 m^2^ during follow-up. The event date was defined as the day of the second qualifying eGFR measurement (<15 mL/min/1.73 m^2^).

### 2.5. Statistical Analysis

The baseline characteristics of the patients are presented as mean ± standard deviation (SD) for continuous variables and as number and percentage for dichotomous variables. Differences in baseline characteristics among groups were assessed as follows: dichotomous variables were analyzed using the chi-square test or Fisher’s exact test; normally distributed continuous variables were analyzed using the *t*-test; and non-normally distributed continuous variables were analyzed using the Mann–Whitney U test.

For descriptive visualization of longitudinal kidney function trajectories, eGFR measurements were grouped into 15-day intervals. When multiple measurements were available for the same participant within an interval, they were averaged within that participant before calculation of the group mean. Absolute eGFR and ΔeGFR values were presented as mean ± standard error. ΔeGFR was calculated as the eGFR at each measurement minus the eGFR on the index day. Group differences at each interval were assessed using Welch’s *t*-test without adjustment for multiple testing.

The eGFR slopes in each group were evaluated using simple linear regression models and LMM. The index day was defined as the reference day, and time was expressed in years. To minimize the influence of short-term fluctuations around the index day, eGFR measurements obtained 0–29 days after the index day were excluded, whereas all measurements before the index day were retained. To estimate distinct slopes in the pre- and post-TAVR periods, eGFR slopes were modeled using a piecewise two-slope specification with separate time variables for the pre- and post-TAVR periods. In the LMM analysis, repeated eGFR measurements within participants were accounted for using participant-specific random intercepts and random slopes for both time components. To improve numerical stability and reduce convergence problems, correlations among the random effects were not estimated. Differences in eGFR slopes by CKD status were assessed by including interaction terms between CKD status and each time component. Group differences were expressed as contrasts and calculated as the slope in patients with CKD minus that in patients without CKD. Within each group, the time differences were estimated as the slope of post-TAVR minus that of pre-TAVR. We additionally analyzed the results from three models: the unadjusted model included time variables, CKD status, and their interaction terms; Model 1 additionally adjusted for age and sex; and Model 2 further adjusted for hemoglobin, N-terminal pro-B-type natriuretic peptide (NT-proBNP), proteinuria, diuretic use, and mineralocorticoid receptor antagonist (MRA) use. The estimated mean eGFR slopes and 95% confidence intervals (CIs) were derived using the estimated marginal trends. The LMMs included all available eligible eGFR measurements from participants included in each model.

Kidney outcome-free survival was estimated using the Kaplan–Meier method and compared between groups using the log-rank test. All analyses were conducted using IBM SPSS Statistics version 26.0 for Windows (IBM Corp., Armonk, NY, USA) and R software version 4.5.1. A *p* value of <0.05 was considered statistically significant.

## 3. Results

### 3.1. Baseline Patient Characteristics

**Figure 1:**
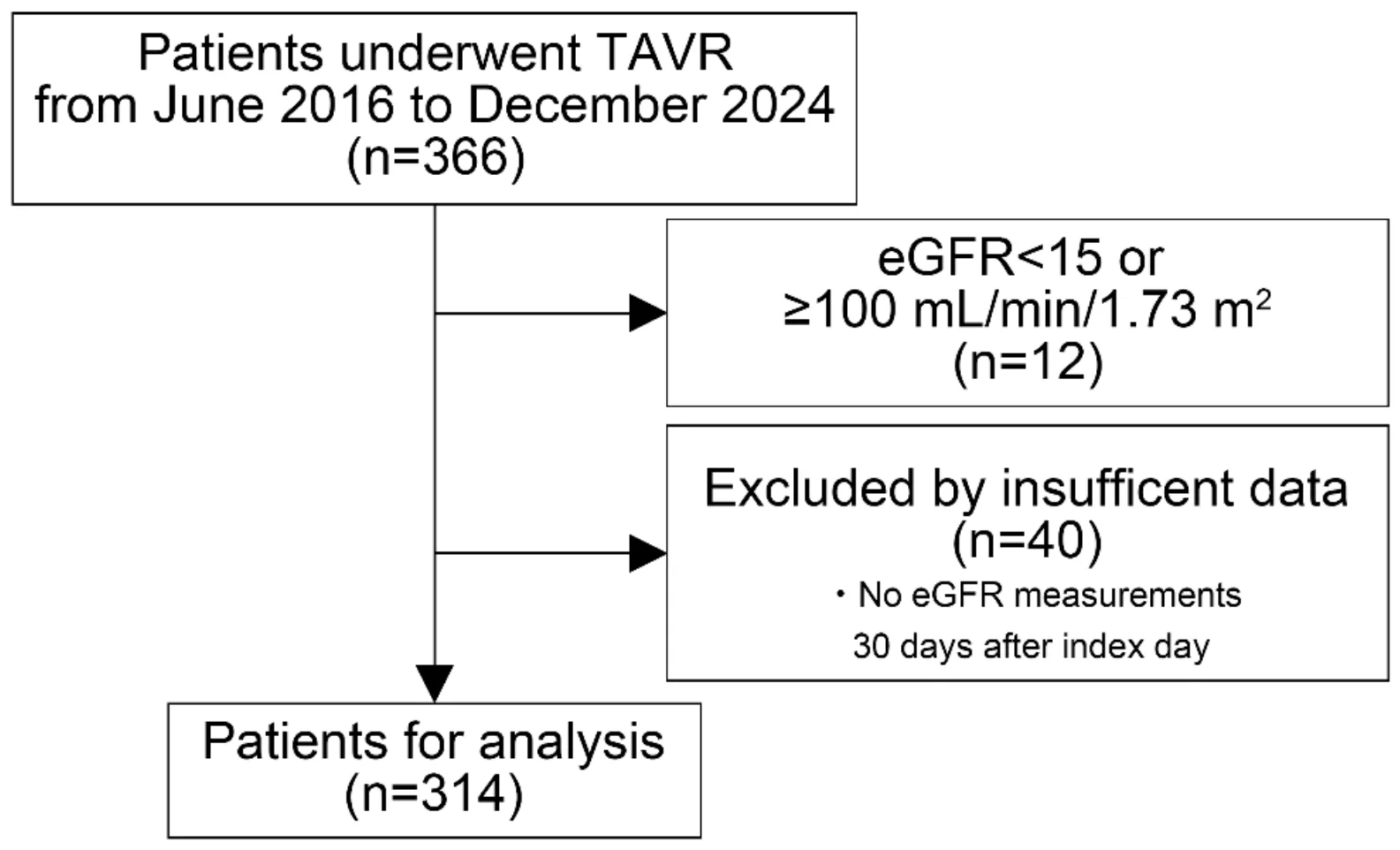
Study flowchart.

Overall, 366 patients who underwent TAVR were enrolled during the study period. Twelve patients were excluded because of advanced CKD stage 5 or eGFR ≥ 100 mL/min/1.73 m^2^. Forty patients were also excluded because no eGFR measurement was available ≥30 days after the index day. In total, 314 patients were included in the analysis, and the study flowchart is summarized in Figure 1.

The median follow-up period was 2.0 years. Among the included patients, 93 (29.6%) were classified as not having CKD and 221 (70.4%) as having CKD. Baseline characteristics stratified by CKD status are shown in Table 1.

Patients without CKD were slightly younger than those with CKD (mean age [standard deviation (SD)] years, 83.6 [4.4] vs. 85.8 [4.5], *p* < 0.01) and had higher baseline kidney function (mean eGFR [SD] mL/min/1.73 m^2^; 71.0 [9.0] vs. 41.5 [11.1]; *p* < 0.001). There were no obvious differences in concomitant comorbidities between the groups. In the laboratory data, group differences were observed for hemoglobin (12.0 [1.8] g/dL in patients without CKD vs. 11.4 [1.8] g/dL in those with CKD; *p* < 0.05) and NT-proBNP (2147.5 [3663.8] pg/mL in patients without CKD vs. 4199.7 [7286.6] pg/mL in those with CKD; *p* < 0.001). A higher rate of proteinuria was observed in patients with CKD than in those without. No differences were found in the echocardiographic data, except for the aortic valve area (AVA, cm^2^) and indexed AVA (cm^2^/m^2^). Medication usage in each group is shown in Table 1, and the use of diuretics and MRAs was higher in patients with CKD than in those without CKD.

### 3.2. Kidney Function Trajectories in Pre- and Post-TAVR Among Patients With or Without CKD

**Figure 2:**
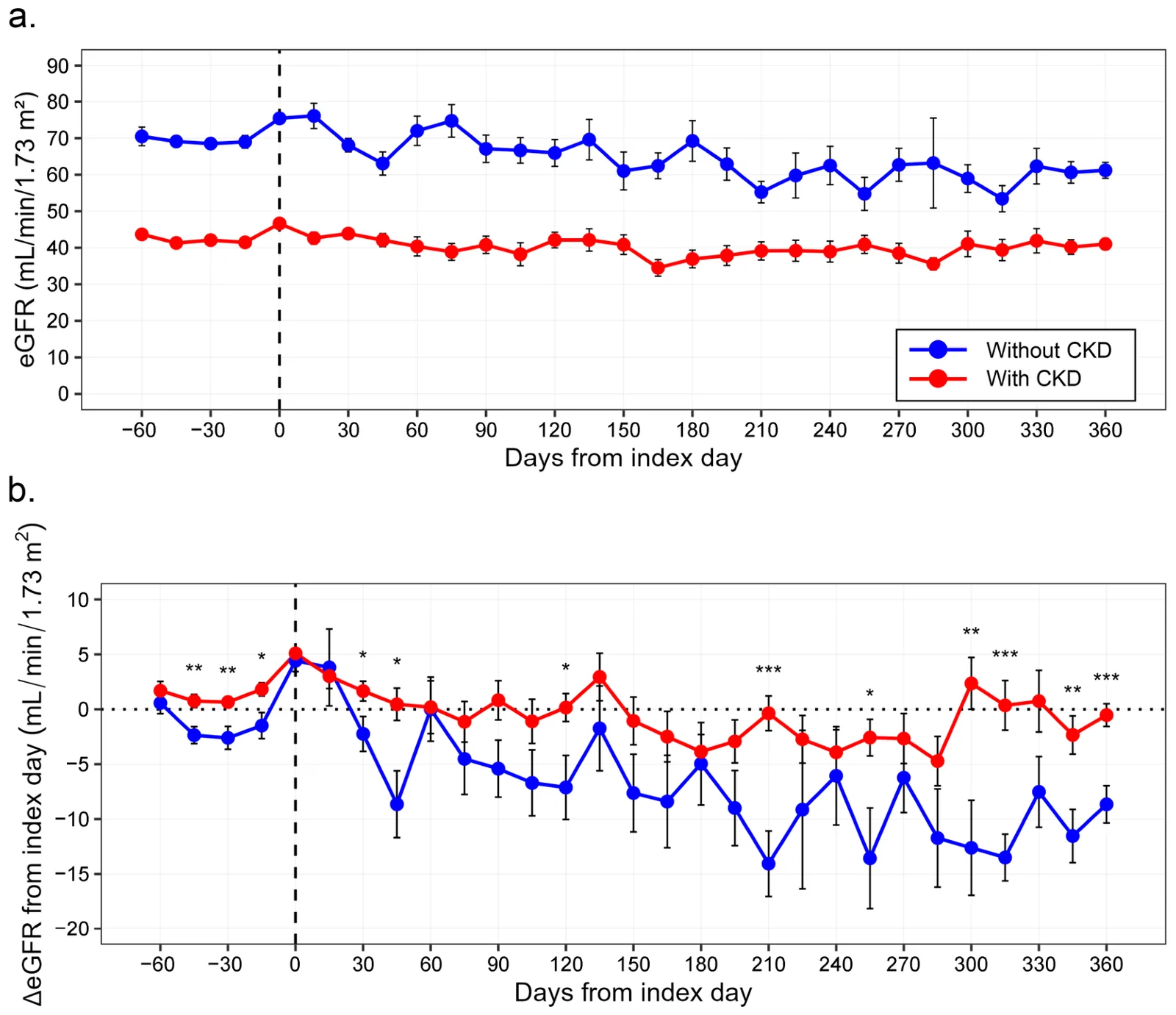
Kidney function trajectories pre- and post-TAVR according to CKD status. (**a**) Mean absolute eGFR values at each plotted time point according to CKD status. (**b**) Mean changes in eGFR from the index day (ΔeGFR) at each plotted time point according to CKD status. Error bars indicate standard errors. The dashed vertical line indicates the index day. Asterisks indicate between-group differences assessed using Welch’s *t*-test at each time point: * *p* < 0.05, ** *p* < 0.01, and *** *p* < 0.001.

Kidney function trajectories pre- and post-TAVR in patients with and without CKD are shown in Figure 2.

During the 1-year follow-up after TAVR, eGFR in patients without CKD gradually declined from a mean [standard error (SE)] of 75.4 [1.3] to 61.2 [2.2] mL/min/1.73 m^2^, whereas eGFR in patients with CKD remained relatively stable, decreasing from a mean [SE] of 46.6 [0.9] to 41.0 [1.6] mL/min/1.73 m^2^ (Figure 2a). Changes in ΔeGFR pre- and post-TAVR in patients with and without CKD are shown in Figure 2b. During the early post-TAVR period, ΔeGFR showed a transient increase in both groups. However, ΔeGFR in patients without CKD gradually declined, whereas it remained stable in patients with CKD during the later post-TAVR period. Notably, differences in ΔeGFR between the groups were significant in the later post-TAVR period.

### 3.3. eGFR Slopes Among Patients With or Without CKD Using a Simple Linear Regression or an LMM

**Figure 3:**
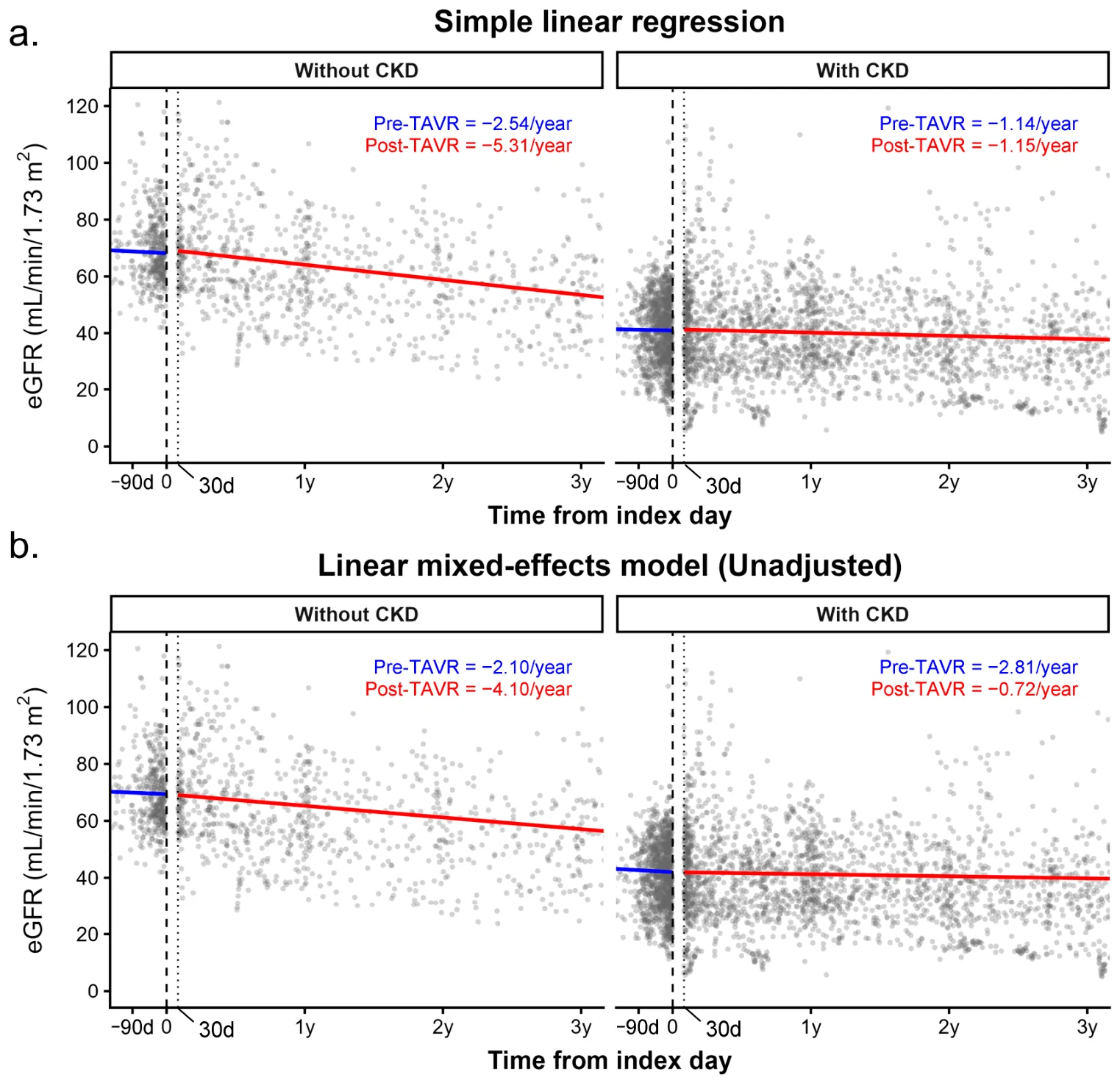
eGFR slopes in pre- and post-TAVR according to CKD status. (**a**) Simple linear regression models. (**b**) Linear mixed-effects models (Unadjusted). eGFR measurements obtained within 0–29 days after the index day were excluded from the post-TAVR slope analysis. The dashed and dotted vertical lines in each panel represent the index day and 30 days after the index day. Grey dots represent individual eGFR measurements, blue lines represent fitted pre-TAVR slopes, and red lines represent post-TAVR slopes. Values shown in each panel are estimated annual eGFR slopes in mL/min/1.73 m^2^/year.

To analyze repeated eGFR measurements, eGFR slopes among patients with or without CKD, which describe trajectories of eGFR decline over time, were calculated using a simple linear regression or a linear mixed-effects model (Figure 3).

In the simple linear regression analysis, patients without CKD had eGFR slopes of −2.54 mL/min/1.73 m^2^/year during the pre-TAVR period and −5.31 mL/min/1.73 m^2^/year during the post-TAVR period. In contrast, patients with CKD had eGFR slopes of −1.14 and −1.15 mL/min/1.73 m^2^/year during the pre- and post-TAVR periods, respectively. During both periods, eGFR declined more slowly in patients with CKD than in those without. Consistent results were obtained using the linear mixed-effects model, as shown in Figure 3b. Additionally, in the fully adjusted linear mixed-effects analysis, the mean annual eGFR slope during the pre-TAVR period among patients without CKD was −2.11 mL/min/1.73 m^2^/year (95% CI, −3.34 to −0.89; *p* < 0.001) compared with −2.83 mL/min/1.73 m^2^/year (95% CI, −3.65 to −2.01; *p* < 0.001) among those with CKD; there were no significant group differences in eGFR slope during the pre-TAVR period. In the post-TAVR period, the mean annual eGFR slope among patients without CKD was −4.02 mL/min/1.73 m^2^/year (95% CI, −5.68 to −2.35; *p* < 0.001) compared with −0.83 mL/min/1.73 m^2^/year (95% CI, −2.04 to 0.39; *p* = 0.182) among those with CKD; the decline in eGFR was significantly slower in patients with CKD than in those without CKD. For the time difference in eGFR slopes between pre- and post-TAVR, the difference among patients without CKD was −1.90 mL/min/1.73 m^2^/year (95% CI, −4.03 to 0.22; *p* = 0.08), whereas the difference among patients with CKD was 2.01 mL/min/1.73 m^2^/year (95% CI, 0.51 to 3.50; *p* < 0.01), as shown in Table 2.

## 4. Discussion

This single-center retrospective cohort study showed that the annual decline in eGFR was relatively slower in patients with CKD than in those without. Specifically, among patients with CKD, the eGFR slope improved significantly after TAVR. Although patients without CKD showed a greater decline in eGFR slope after TAVR, their mean eGFR remained above 60 mL/min/1.73 m^2^ at 1 year, suggesting that the risk of progression to kidney failure remained relatively low. Therefore, TAVR may be performed without excessive concern regarding progression to severely reduced kidney function, even in older adults at high risk for atherosclerotic disease.

Kidney function is closely linked to systemic perfusion and cardiac output. Hemodynamic dysfunction caused by AS may compromise kidney perfusion, whereas worsening kidney function may accelerate AS progression [10]. This reciprocal pathophysiological process is referred to as cardiorenal syndrome [19,20], and TAVR may help interrupt this vicious cycle in patients with AS. TAVR is expected to increase cardiac output and kidney perfusion and reduce venous pressure, thereby improving kidney function. Nonetheless, despite these potential benefits of TAVR for kidney function, its impact on postprocedural kidney function in real-world clinical practice remains controversial, particularly over the long term. In a single-center retrospective cohort study by Steinmetz et al. [21], patients with CKD who underwent TAVR had stable kidney function at 1 year, with mean eGFR increasing from 41.7 to 42.9 mL/min/1.73 m^2^. Another study by Soodi et al. reported a modest decrease in mean eGFR from 47.6 to 43.2 mL/min/1.73 m^2^ at 3 years after TAVR among patients with CKD [22]. Our findings regarding the eGFR slope among patients with CKD after TAVR, estimated using an LMM, are consistent with those of these previous reports. Although patients undergoing TAVR are exposed to several potential risk factors for kidney injury, including contrast media use, periprocedural hypotension, and cholesterol embolization, our findings suggest that TAVR does not necessarily lead to excessive deterioration of kidney function or progression to kidney failure.

Unlike patients with CKD, those without CKD appeared to derive less hemodynamic benefit for the kidneys from TAVR, and their post-TAVR eGFR trajectory showed a relatively steeper decline. Several possible explanations can be considered. First, patients without CKD had a higher baseline eGFR and may, therefore, have had a smaller hemodynamic component of kidney dysfunction attributable to severe AS. Accordingly, any improvement in kidney perfusion after relief of AS with TAVR may have been limited in this group. Second, although eGFR measurements obtained within the first 30 days after TAVR were excluded from the slope analysis, relatively preserved eGFR values early after TAVR were followed by lower values during subsequent follow-up. Therefore, summarizing this nonlinear trajectory as a linear post-TAVR slope may have resulted in a more negative estimated slope, particularly in patients without CKD. Third, the use of renoprotective medications has differed between the groups. In this cohort, the proportions of RAASi and MRAs at baseline were relatively higher in patients with CKD than in those without CKD. These medications might have contributed to the preservation of kidney function in the CKD group. Finally, patients with CKD may have been more likely to be lost to follow-up than those without CKD, potentially introducing attrition bias by preferentially retaining patients with a more favorable clinical status.

Previous studies have shown heterogeneous kidney function trajectories after TAVR, irrespective of baseline CKD status [11,23,24]. For example, one study [12] reported that 36.8% of patients showed an improvement in eGFR of at least 10% at 1 month after TAVR, whereas 26.1% showed a decline in eGFR of at least 10%. These findings suggest that kidney function after TAVR varies among patients. Some patients experience improvement, whereas others remain stable or show worsening kidney function, depending on individual clinical characteristics. Indeed, because the underlying causes of CKD could not be determined in this study, differences in primary kidney disease may have contributed to the heterogeneity in post-TAVR kidney function trajectories. However, given the advanced age of the patients, the presence of systemic atherosclerotic disease, including calcific aortic valve disease, and the generally mild degree of proteinuria, nephrosclerosis was considered the predominant underlying kidney disease in this population. The number of patients without CKD was relatively small, which may have resulted in greater variability and limited precision in estimating the post-TAVR eGFR slopes in this group. Importantly, despite the relatively steeper decline in the eGFR slope among patients without CKD, no progression to kidney failure was observed in this population, and progression to kidney failure was observed in only 4.5% of patients with CKD during a 3-year observational period (Supplementary Figure S1). TAVR may appear to be associated with excessive deterioration of kidney function, leading to kidney failure. However, our findings suggest that TAVR may be performed relatively safely with respect to kidney outcomes.

This study has some limitations: (1) the generalizability of our findings may be limited because this was a single-center, retrospective cohort study; (2) the frequency and timing of eGFR measurements varied among patients, particularly in the post-TAVR period; (3) potential biases related to informative missingness or differences in follow-up intensity cannot be fully excluded; (4) detailed information on medication changes and periprocedural factors, such as contrast media exposure, antibiotic use, intravenous fluid administration, procedural time, and postprocedural complications, were not fully incorporated into the analysis; and (5) other kidney biomarkers, such as proteinuria, could not be assessed longitudinally because these data were not consistently available during follow-up.

## 5. Conclusions

In this study, post-TAVR eGFR decline was attenuated in patients with CKD relative to those without, and kidney failure was uncommon. The findings provide clinically relevant information regarding long-term kidney function after TAVR, including in patients with CKD. Further prospective studies are warranted to confirm these findings.

## Figures and Tables

**Table 1:** Baseline clinical characteristics.

	All	Without CKD	With CKD	*p* Value
Number of patients (n)	314	93	221	
Age (y)	85.2 (4.6)	83.6 (4.4)	85.8 (4.5)	<0.01
Sex (men, %)	36.3	40.9	34.4	0.276
BMI (kg/m^2^)	23.0 (4.0)	23.4 (4.4)	22.9 (3.9)	0.330
Pulse rate (bpm)	72.1 (12.0)	72.0 (11.7)	72.2 (12.2)	0.902
Systolic blood pressure (mmHg)	124.5 (21.3)	124.4 (18.5)	124.6 (22.4)	0.929
Diastolic blood pressure (mmHg)	67.0 (13.1)	68.6 (11.7)	66.3 (13.7)	0.157
Comorbidities				
Hypertension (yes, %)	81.8	79.6	82.8	0.497
Hypercholesterolemia (yes, %)	65.0	65.6	64.7	0.881
Diabetes mellitus (yes, %)	31.5	32.3	31.2	0.857
Smoking (yes, %)	33.8	35.5	33.0	0.675
Laboratory data				
Hemoglobin (g/dL)	11.6 (1.8)	12.0 (1.8)	11.4 (1.8)	<0.05
Platelet (×10^4^/µL)	19.8 (10.2)	20.7 (6.1)	19.4 (11.5)	0.284
eGFR (mL/min/1.73 m^2^)	50.3 (17.1)	71.0 (9.0)	41.5 (11.1)	<0.001
Total protein (g/dL)	7.0 (0.6)	7.0 (0.6)	7.0 (0.6)	0.784
NT-proBNP (pg/mL)	3591.9 (6491.9)	2147.5 (3663.8)	4199.7 (7286.6)	<0.001
Echocardiographic data				
Ejection fraction (%)	59.6 (9.7)	61.1 (8.8)	58.9 (10.0)	0.075
Septal wall thickness (mm)	12.6 (1.7)	12.8 (1.6)	12.6 (1.7)	0.174
Aortic valve area (cm^2^)	0.62 (0.17)	0.65 (0.19)	0.61 (0.15)	<0.05
Indexed aortic valve area (cm^2^/m^2^)	0.42 (0.11)	0.44 (0.14)	0.41 (0.10)	<0.05
Peak aortic jet velocity (m/s)	4.6 (0.7)	4.6 (0.7)	4.5 (0.7)	0.449
Mean aortic valve gradient (mmHg)	48.9 (17.0)	50.1 (18.3)	48.4 (16.5)	0.415
Max aortic valve gradient (mmHg)	85.3 (46.9)	84.6 (25.5)	85.6 (53.5)	0.862
Medications				
Beta-blockers (yes, %)	40.8	33.3	44.3	0.070
Statins (yes, %)	59.2	63.4	57.5	0.325
RAASi (yes, %)	54.5	49.5	56.6	0.249
Diuretics (yes, %)	49.4	30.1	57.5	<0.001
MRAs (yes, %)	24.5	17.2	28.1	<0.05
SGLT2i (yes, %)	7.0	7.5	6.8	0.815
Urinary protein				
(−) or (±) (%)	80.3	90.3	75.9	<0.05
(1+) (%)	14.9	7.5	18.1	
(2+) (%)	3.2	1.1	4.2	
(3+) (%)	0.6	0.0	0.9	
(4+) (%)	1.0	1.1	0.9	

Data are presented as mean (standard deviation) or percentage. CKD, chronic kidney disease; BMI, body mass index; eGFR, estimated glomerular filtration rate; NT-proBNP, N-terminal pro-B-type natriuretic peptide; RAASi, renin–angiotensin–aldosterone system inhibitor; MRA, mineralocorticoid receptor antagonist; SGLT2i, sodium–glucose cotransporter 2 inhibitor.

**Table 2:** eGFR slopes in pre- and post-TAVR according to CKD status using linear mixed-effects models.

eGFR Slope (pre-TAVR)	Unadjusted Model	*p* Value	Model 1	*p* Value	Model 2	*p* Value
Without CKD	−2.10 [−3.31, −0.89]	<0.001	−2.10 [−3.31, −0.88]	<0.001	−2.11 [−3.34, −0.89]	<0.001
With CKD	−2.81 [−3.61, −2.00]	<0.001	−2.81 [−3.62, −2.00]	<0.001	−2.83 [−3.65, −2.01]	<0.001
Group difference	−0.71 [−2.16, +0.75]	0.342	−0.71 [−2.17, +0.75]	0.338	−0.72 [−2.19, +0.75]	0.339
eGFR slope (post-TAVR)	Unadjusted model	*p* value	Model 1	*p* value	Model 2	*p* value
Without CKD	−4.10 [−5.76, −2.44]	<0.001	−4.09 [−5.75, −2.43]	<0.001	−4.02 [−5.68, −2.35]	<0.001
With CKD	−0.72 [−1.92, +0.48]	0.237	−0.75 [−1.95, +0.45]	0.219	−0.83 [−2.04, +0.39]	0.182
Group difference	+3.38 [+1.64, +5.12]	<0.001	+3.34 [+1.60, +5.08]	<0.001	+3.19 [+1.44, +4.94]	<0.001
Time difference (post−pre)	Unadjusted model	*p* value	Model 1	*p* value	Model 2	*p* value
Without CKD	−2.00 [−4.11, +0.11]	0.06	−2.00 [−4.11, +0.12]	0.06	−1.90 [−4.03, +0.22]	0.08
With CKD	+2.08 [+0.61, +3.56]	<0.01	+2.06 [+0.58, +3.53]	<0.01	+2.01 [+0.51, +3.50]	<0.01

Values are estimated annual eGFR slopes [95% confidence intervals], expressed as mL/min/1.73 m^2^/year. Group differences were calculated as the slope in patients with CKD minus that in patients without CKD. Time differences were calculated as the post-TAVR slope minus the pre-TAVR slope. Model 1 was adjusted for age and sex. Model 2 was additionally adjusted for hemoglobin, NT-proBNP, proteinuria, diuretic use, and mineralocorticoid receptor antagonist use.

## Data Availability

The data are not publicly available due to privacy and ethical restrictions. The data supporting the findings of this study are available from the corresponding author upon reasonable request and in accordance with institutional and ethical requirements.

## Supplementary Materials

The following supporting information can be downloaded at https://www.mdpi.com/article/10.3390/jcm15197656/s1, Figure S1: Kidney outcome within 3 years post-TAVR according to CKD status. (a) Incidence of kidney outcome in patients with and without CKD. The p-value was calculated using Fisher’s exact test. (b) Kaplan–Meier curves for kidney outcome-free survival in patients with and without CKD. Tick marks indicate censored observations, and numbers below the graph indicate the number of patients at risk. The p-value was calculated using the log-rank test. CKD, chronic kidney disease.

## Abbreviations

The following abbreviations are used in this manuscript:

AKI	Acute Kidney Injury
AS	Aortic Stenosis
AVA	Aortic Valve Area
AVR	Aortic Valve Replacement
BMI	Body Mass Index
CI	Confidence Interval
CKD	Chronic Kidney Disease
eGFR	Estimated Glomerular Filtration Rate
LMM	Linear Mixed-effects Model
MRA	Mineralocorticoid Receptor Antagonist
NT-proBNP	N-Terminal pro-B-type Natriuretic Peptide
RAASi	Renin–Angiotensin–Aldosterone System Inhibitor
SAVR	Surgical Aortic Valve Replacement
SD	Standard Deviation
SE	Standard Error
SGLT2i	Sodium–Glucose cotransporter 2 Inhibitor
TAVR	Transcatheter Aortic Valve Replacement
