# Supplementary material for: Kidney Function Trajectories in Patients with and Without Chronic Kidney Disease Undergoing Transcatheter Aortic Valve Replacement: A Single-Retrospective Cohort Study
Source: J Clin Med. 2026 Oct 2;15(19):7656. doi: 10.3390/jcm15197656 (PMC13666079; doi:10.3390/jcm15197656)
Supplement: Supplementary file 1 [file jcm-15-07656-s001.zip › jcm-4533430-supplementary.pdf]

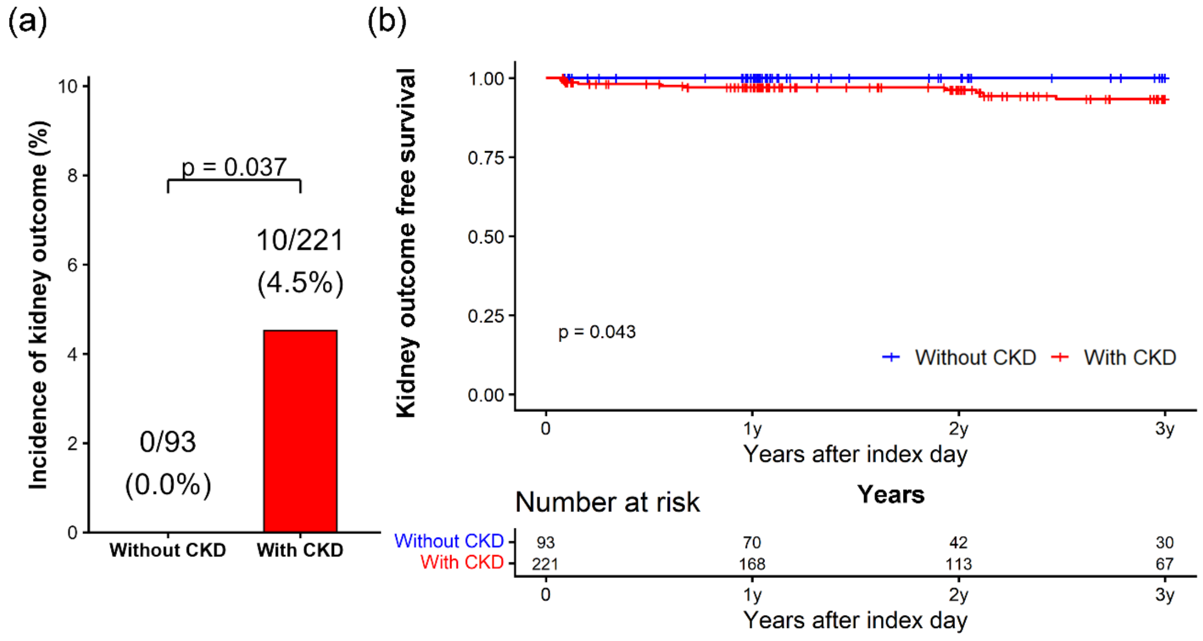

Supplementary Figure S1. Kidney outcome within 3 years post-TAVR according to CKD status. (a) Incidence of kidney outcome in patients with and without CKD. The p-value was calculated using Fisher's exact test. (b) Kaplan–Meier curves for kidney outcome-free survival in patients with and without CKD. Tick marks indicate censored observations, and numbers below the graph indicate the number of patients at risk. The p-value was calculated using the log-rank test. CKD, chronic kidney disease.
